# Hyperspectral reflectance sensing to assess the growth and photosynthetic properties of wheat cultivars exposed to different irrigation rates in an irrigated arid region

**DOI:** 10.1371/journal.pone.0183262

**Published:** 2017-08-22

**Authors:** Salah El-Hendawy, Nasser Al-Suhaibani, Wael Hassan, Mohammad Tahir, Urs Schmidhalter

**Affiliations:** 1 Department of Plant Production, College of Food and Agriculture Sciences, King Saud University, Riyadh, Saudi Arabia; 2 Department of Agronomy, Faculty of Agriculture, Suez Canal University, Ismailia, Egypt; 3 Department of Agricultural Botany, Faculty of Agriculture, Suez Canal University, Ismailia, Egypt; 4 Department of Biology, Quwayiyah College of Science and Humanities, Shaqra University, Shaqra, Saudi Arabia; 5 Chair of Plant Nutrition, Department of Plant Sciences, Technical University of Munich, Freising-Weihenstephan, Germany; Estacion Experimental del Zaidin, SPAIN

## Abstract

Simultaneous indirect assessment of multiple and diverse plant parameters in an exact and expeditious manner is becoming imperative in irrigated arid regions, with a view toward creating drought-tolerant genotypes or for the management of precision irrigation. This study aimed to evaluate whether spectral reflectance indices (SRIs) in three parts of the electromagnetic spectrum ((visible-infrared (VIS), near-infrared (NIR)), and shortwave-infrared (SWIR)) could be used to track changes in morphophysiological parameters of wheat cultivars exposed to 1.00, 0.75, and 0.50 of the estimated evapotranspiration (ETc). Significant differences were found in the parameters of growth and photosynthetic efficiency, and canopy spectral reflectance among the three cultivars subjected to different irrigation rates. All parameters were highly and significantly correlated with each other particularly under the 0.50 ETc treatment. The VIS/VIS- and NIR/VIS-based indices were sufficient and suitable for assessing the growth and photosynthetic properties of wheat cultivars similar to those indices based on NIR/NIR, SWIR/NIR, or SWIR/SWIR. Almost all tested SRIs proved to assess growth and photosynthetic parameters, including transpiration rate, more efficiently when regressions were analyzed for each water irrigation rate individually. This study, the type of which has rarely been conducted in irrigated arid regions, indicates that spectral reflectance data can be used as a rapid and non-destructive alternative method for assessment of the growth and photosynthetic efficiency of wheat under a range of water irrigation rates.

## Introduction

Water shortage is one of the main abiotic factors that limit the productivity of staple crops in arid and semi-arid regions. Additionally, the climatic conditions in these regions are typically characterized by sparse and highly variable rainfall, together with high temperatures and potential evapotranspiration. Furthermore, at least 80% of the cropping area in these regions is irrigated and therefore the agricultural sector consumes on average approximately 75% of the total available water [1]. Consequently, achieving maxim production per unit of water irrigation applied remains a major objective for agricultural research in these regions, through gradually replacing the paradigm of full irrigation with deficit irrigation [2, 3].

A better understanding of the responses of morphophysiological parameters to water deficit stress will provide useful guidelines to plant agronomists on how to maximize and sustain crop production and water-use efficiency when water shortages are the rule rather than the exception. For plant physiologists, elucidating such responses will enable identification of the parameters that can be used as screening criteria for drought tolerance. It will also promote an understanding of the mechanisms underlying drought tolerance, thereby enabling plant breeders to incorporate these parameters into breeding programs to improve or create new drought-tolerant genotypes, and to identify the genes controlling these parameters.

Several important morphophysiological parameters, such as dry matter accumulation, leaf expansion, green leaf area, leaf gas exchange, stomatal behavior, and transpiration rate, are components of a cascade of plant responses to water deficit stress [4, 5]. It is well established that the earliest responses to water deficit stress involve minimizing stomatal conductance (*Gs*) to lower the amount of water loss through transpiration and enhancing water-use efficiency [6]. However, by lowering substomatal CO_2_ concentration, this drought-adaptive mechanism leads to a decrease in photosynthetic rate (*Pn*) during the early stage of mild and moderate water deficit stresses [7, 8]. Severe water deficit stress also inhibits plant photosynthesis through non-stomatal factors by causing changes in the accumulation and distribution of photosynthetic pigments [9]. Additionally, the decrease in stomatal aperture size under prolonged water deficit stress is associated with adjustments of leaf area at the whole-plant level. The leaf area of the canopy is adjusted either through the earlier senescence of older leaves or via a reduction in leaf development. This drought-avoidance mechanism leads to decreased transpiration rate (*E*) but also results in a decrease in intercepted radiation, which ultimately leads to a reduction in biomass accumulation [4]. Accordingly, in order to study the responses of growth and photosynthetic efficiency of plants to water deficit stress, it is important to monitor these responses at the canopy scale because the growth and productivity of plants are dependent on photoassimilates produced at the whole-plant level [10].

Given that there is an integrated response of morphophysiological parameters, i.e., biomass accumulation, leaf area, *Pn*, *Gs*, and *E*, to water deficit stress, and that this stress can generate a variety of plant responses, which can be intensified or act synergistically or antagonistically [11], the drought tolerance of genotypes should be assessed by simultaneously measuring multiple morphophysiological parameters. Unfortunately, there are currently no traditional methods whereby multiple morphophysiological parameters can be measured simultaneously. Furthermore, measurement of these parameters based on plant sampling techniques is generally tedious, destructive, and time consuming, and often inappropriate for tracking the dynamics of physiological parameters or for fulfilling the requirement for real-time evaluation of morphological parameters [12, 13]. Importantly, although the parameters related to photosynthetic efficiency (*Pn*, *Gs*, and *E*) can be simultaneously measured in a rapid and non-destructive manner using a portable gas exchange system, this method provides information on the photosynthetic status solely on a single leaf. Because the age and position of leaves in a single plant or within the canopy have distinct morphological, anatomical, and physiological characteristics, this generates huge variations in photosynthetic efficiency within the canopy [14–17]. Measurements of photosynthetic parameters in wheat are generally made using the flag leaf or fully expanded penultimate leaves, which have a high rate of photosynthesis [18]. Therefore, measurements of photosynthesis parameters based on such leaves are probably unrepresentative of most leaves in the canopy. Accordingly, measurements based solely on single leaves do not accurately reflect the photosynthetic behavior of an entire canopy, particularly when different wheat genotypes are evaluated under different levels of water stress. In addition, although the measurement of shoot biomass does not require special expertise, as does the measurement of photosynthesis parameters, such measurements can be influenced by human fatigue and bias that are often the consequence of high-volume sample processing, such as harvesting, drying, and weighing, which makes repeated measurements on the same plant sample impossible. Consequently, a more efficient, accurate, rapid, and non-destructive alternative method is needed to address the aforementioned drawbacks of traditional methods.

Hyperspectral reflectance sensing technique has been demonstrated to be a valuable and powerful alternative to traditional methods for the simultaneous indirect assessment of hundreds of morphophysiological parameters in a rapid, non-destructive, and consistent manner under different abiotic stresses [1, 19–24]. Such technique is based on the capabilities of plants to reflect and absorb the light or solar radiation at the visible-infrared (VIS, 400–700 nm) the near-infrared (NIR, 700–1300 nm) and the shortwave-infrared (SWIR, 1300–2500 nm) wavelengths depending on biophysical and biochemical characteristics of the canopy, which are sensitive to water deficit stress. These capabilities give spectral reflectance data a great potential for use in detecting and quantifying stress-related plant parameters [25]. To translate these spectral reflectance data into information on vegetation characteristics, different spectral reflectance indices (SRIs) are computed and related to biophysical and biochemical characteristics of the canopy.

Many SRIs have been used to assess different crop parameters under either normal and/or stress conditions. The most widely used SRIs for monitoring and quantifying different crop parameters, particularly those associated with drought stress, such as green and dry biomass, photosynthetic pigment concentration, photosynthetic activity, and plant water status, are near-infrared (NIR)-based indices and/or a combination of visible (VIS)- and NIR-based indices [19, 20, 22, 23, 26–34]. However, there is still a lack of consensus regarding which specific wavelengths of the spectrum are most effective and should be incorporated in SRIs to detect changes in different morphophysiological parameters. For instance, the photochemical reflectance index (PRI), which is based on the VIS spectrum, has been increasingly used to assess changes in plant photosynthetic performance in different plant species at both the leaf and canopy levels [33, 35–37]. The normalized difference vegetation index (NDVI) and water index (WI), which are based on the NIR spectrum, are used extensively to assess green biomass, *Gs*, and the leaf water status, [29, 38–40]. Babar et al. [40] concluded that NIR-based indices were more efficient than other indices in terms of differentiating the dry biomass of different wheat cultivars under irrigated conditions. Working with 368 advanced lines and cultivars of spring wheat and 70 SRIs under three different water regimes, Lobos et al. [22] reported that NIR-based indices proved to be better predictors of growth and production than those based on a combination of the VIS and NIR regions of the spectrum. In *Olea europaea* plants, it was reported [33, 34] that WI tracked leaf *Pn*, *E*, and *Gs* more effectively than the water content relative index (WCRI), the latter of which is based on a combination of wavelengths in shortwave-infrared (SWIR) and NIR regions. In contrast, Ceccato et al. [41], Bayat et al. [42], and Ranjan et al. [43] showed that under four levels of irrigation, the moisture stress index (MSI), the normalized difference infrared index (NDII), the normalized difference water index-1640 (NDWI-1640), and the normalized multi-band drought index (NMDI), which are based on wavelengths in the NIR and SWIR regions of the spectrum, are the best predictor indices for relative leaf water content at the booting growth stage of wheat. Variability in the growing conditions, growth stage, level of drought stress, methods of measurements, and plant species may be among the main reasons explaining the aforementioned conflicting results in spectral reflectance measurements. Therefore, there is still a need to further validate various SRIs for phenotyping drought stress-related plant parameters under a wide range of conditions.

Studies designed to improve our knowledge about the relationship between drought stress-related plant parameters and SRIs have, in most cases, been performed under rain-fed conditions or controlled drought stress conditions using rain-out shelter facilities or potted plants. Because at least 75% of the crop growing area in arid and semi-arid regions is irrigated, the management of deficit water irrigation to increase water productivity has become a major target for plant agronomists and physiologists. To the best of our knowledge, there are few studies that have examined the relationships between SRIs and different morphophysiological parameters under various levels of irrigation [1, 25]. The aims of this study were therefore (1) to compare the growth and photosynthetic properties of three wheat cultivars exposed to different levels of irrigation, (2) to compare the spectral signatures of the canopy of the three cultivars in response to different irrigation rates in three regions of the spectrum (VIS, NIR, and SWIR), and (3) to evaluate whether SRIs could be used to assess the growth and photosynthetic properties of wheat cultivars when subjected to different levels of irrigation in a high-throughput mode.

## Materials and methods

### Plant materials and growth conditions

Three different wheat cultivars, Pavon 76, Sakha 93, and Yecora Rojo, were selected for investigation in this study based on their drought tolerance. The Pavon 76 and Yecora Rojo cultivars have been evaluated previously and identified as drought-tolerant and drought-sensitive, respectively [44]**.** Sakha 93 has also been identified as a drought-tolerant cultivar in previous studies [1, 39].

The three cultivars were grown under field conditions at the Dierab Research Station of the Department of Plant Production, College of Food and Agriculture Sciences, King Saud University, Riyadh, Saudi Arabia (24°25′N, 46°34′E; 400 m a.s.l.) during the 2015/2016 and 2016/2017 growing seasons. This research station is characterized by a typical arid climate. The temperature and precipitation during the entire period of wheat growth were in the ranges of 9.9–35.2°C and 5–28 mm, respectively. The soil texture is a sandy loam throughout its profile (76.1% sand, 15.4% silt, and 8.5% clay), with a field capacity and wilting point of 0.148 and 0.090 m^3^ m^-3^, respectively, and a soil bulk density of 1.51 g cm^-3^. The pressure plate technique was used to determine the water content at field capacity and wilting point as described by [45].

### Experimental design, crop management, and irrigation treatments

The field experiments were conducted as a randomized complete block split-plot design and replicated three times, with the three levels of irrigation and the three wheat cultivars being kept in the main plots and subplots, respectively. Each subplot consisted of ten 4.0-m-long rows spaced 15 cm apart (6.0 m^2^ in total area). Each cultivar was planted at a seeding rate of 150 kg ha^-1^. A total of 180 kg N ha^-1^ and 31.0 kg P_2_O_5_ ha^-1^ were applied as ammonium nitrate and monocalcium phosphate, respectively. Nitrogen fertilizer was applied at the seedling, stem elongation, and booting stages, whereas the entire amount of phosphorus was applied basally before sowing. Prophylactic application of herbicides and fungicides was also undertaken to control weed infestations and diseases, respectively.

To create a range of water deficit stress, three water application rates (1.00, 0.75, and 0.50 of the estimated crop evapotranspiration (ETc) were used in this study. The amount of irrigation water applied for 1.00 ETc was calculated using the following equation (Eq 1):

ETc=ETo×Kc, (1)

where ETc is the water requirement for wheat crops (mm day^-1^), ETo is the reference evapotranspiration (mm day^-1^), and Kc is the crop coefficient.

The reference evapotranspiration was calculated using the FAO-56 Penman-Monteith equation [46] based on meterological data obtained from weather stations located within 200 m of the experimental site.

The values of Kc for wheat recommended by FAO-56 [46] were used after adaptation to the conditions of the study area. The Kc values must be adjusted in different areas, where minimum relative humidity differs from 45% and wind velocity measured at 2 m height is sometimes greater or less than 2 m s^-1^. The Kc values for the mid- and final wheat growth stages were adjusted using the following equation (Eq 2):

$$Kc=Kc(table)+\left[\left(0.04(U_{2}-2)-0.004(RH_{\min}-45\right)\right]{\left(h/3\right)}^{0.3},$$ (2)

where Kc (table) is the standard Kc values recommended by FAO-56 [46], U_2_ is the daily wind speed at 2 m height (m s^-1^), RH_min_ is the minimum relative humidity, and h is the plant height of wheat for each growth stage (m).

After the water requirement for the control treatment (1.00 ETc) was calculated, the amount of water was reduced to 75% and 50% for the 0.75 and 0.50 ETc water deficit treatments, respectively. Averaged over the two growing seasons, the total amounts of irrigation water applied were 6050, 4537.5, and 3025.0 m^3^ ha^-1^ for the 1.00, 0.75, and 0.50 ETc treatments, respectively. Irrigation was scheduled based on class A pan. The irrigation was carried out after the amount of evaporation water from class A pan accumulated to about 80 mm [47]. The irrigation water was applied via a surface irrigation system. This system had one water-emitting tube in each plot and was equipped with manual control valves and a discharge gauge to deliver constant and equal amounts of water to each plot.

### Measurements

#### Growth and photosynthetic parameters

Parameters for growth [green leaf number per plant (GLN), green leaf area per plant (GLA), and total shoot dry weight per plant (TDW)] and photosynthesis [net photosynthesis rate (*P*_*n*_), stomatal conductance (*Gs*), and transpiration rate (*E*)] were measured at the anthesis growth stage. Growth parameters were determined for 20 plants collected randomly from each subplot. The green leaves were separated from plant samples, counted, and their leaf area measured using a leaf area meter (LI 3100; LI-COR Inc., Lincoln, NE, USA). All parts of plant samples (green leaves, completely brown leaves, stem, and spikes) were collected together and then dried at 75°C in a forced-air oven for 72 h, and then weighed to obtain the total shoot dry weight per plant.

All photosynthetic parameters were measured on the central section of the second fully expanded leaf from the top of the plant using a portable gas exchange system (Li-6400; Li-COR Inc., Lincoln, NE, USA) between 10.00 and 15.00 h. During the measurements, the leaf chamber was set to a leaf temperature and a CO_2_ concentration of 25°C and 400 ppm, respectively.

#### Canopy spectral reflectance and spectral reflectance indices (SRIs)

In parallel with growth and photosynthetic parameter measurements, canopy spectral reflectance was measured using a portable field spectrometer (ASD Fieldspec Pro; Analytical Spectral Devices Inc., Boulder, CO, USA), which enables detection of the spectral signature of the canopy in the range 350–2500 nm with a bandwidth of 1.0 nm [1, 24, 39]. Measurements were made at clear weather under field conditions between 10.00 and 15.00 h (under arid conditions and during flowering until the maturity stage of wheat the wather remains fairly stable during the period of measurements [1, 24, 39]. Before and after reflectance measurements for each sub-plot, the spectrometer was calibrated using a white reference panel covered with a mixture of barium sulfate (BaSO_4_) and white paint in order to avoid problems originating from sun angles during the period of measurement. A fiber optic probe (2.3-mm diameter and 25° full conical angle) was held vertically above the canopy in the nadir position, approximately 80 cm above the canopy, to view ~28 cm^2^ in the center of each sub-plot. To minimize measurement noise and acquire an accurate value, four spectral signatures of the canopy were recorded for each sub-plot at different sites, and the average of the four readings was used to estimate the spectral response. View Spec Pro (ASD) software was used for pre-processing reflectance spectra and to prepare for calculations of the different SRIs. We calculated 31 SRIs that combined wavelengths from three parts of the spectrum (VIS, NIR, and SWIR regions). The full names and abbreviations of these SRIs are listed in **Tables 1 and 2.**

**Table 1:** Full name and abbreviation (Abbr.) of the spectral reflectance indices (SRI) used in this study and formulated using VIS/VIS, NIR/VIS, and NIR/NIR of the spectrum.

Full name of SRIs and Abbr.	Formula
**VIS/VIS and NIR/VIS spectrum**	
Photochemical reflectance index (PRI)	(R531 –R_570_)/(R_531_ + R_570_)
Normalized phaeophytinization index (NPQ)	(R_415_ –R_435_)/(R_415_ + R_435_)
Modified chlorophyll absorption ratio index (MCARI)	[(R_750_ –R_705_)– 0.2 × (R_750_ –R_550_)] × (R_750_/R_705_)
Green chlorophyll index (GCI)	(R_800_/R_550_)– 1
Carter index 2 (CTR-2)	(R_695_/R_760_)
Pigment specific simple ratio-a (PSSR-a)	(R_800_ /R_680_)
Pigment specific simple ratio-b (PSSR-b)	(R_800_/R_635_)
Pigment specific simple ratio-c (PSSR-c)	(R_800_/R_470_)
Pigment specific normalized difference-a (PSND-a)	(R_800_ –R_680_)/(R_800_ + R_680_)
Pigment specific normalized difference-b (PSND-b)	(R_800_ –R_635_)/(R_800_ + R_635_)
Pigment specific normalized difference-c (PSND-c)	(R_800_ –R_470_)/(R_800_ + R_470_)
Optimized soil adjusted vegetation index (OSAVI)	1.16 × (R_800_ –R_670_)/(R_800_ + R_670_ + 0.16)
Enhanced vegetation index-2 (EVI-2)	2.5 [(R_800_ –R_660_)/(1 + R_800_ + 2.4 × R_660_)]
Modified transformed vegetation index (MTVI)	1.2 × [(1.2 × (R_800_ –R_550_)– 2.5 × (R_670_ –R_550_)]
Structure insensitive pigment index (SIPI)	(R_800_ –R_445_)/(R_800_ –R_680_)
**NIR/NIR spectrum**	
Normalized difference vegetation index (NDVI)	(R_750_ –R_705_) /(R_750_ + R_705_)
Water index (WI)	(R_900_/R_970_)
Normalized water index -3 (NWI-3)	(R_970_ –R_880_)/(R_970_+R_880_)
Normalized different water index-1240 (NDWI-1240)	(R_860_ –R_1240_)/(R_860_ + R_1240_)
Red edge model (REM)	(R_800_/R_700_)– 1

**Table 2:** Full name and abbreviation (Abbr.) of the spectral reflectance indices (SRI) used in this study and formulated using NIR/SWIR and SWIR/SWIR of the spectrum.

Full name of SRIs and Abbr.	Formula
NIR/SWIR and SWIR/SWIR spectrum	
Reciprocal of moisture stress index (RMSI)	(R_860_/R_1650_)
Shortwave infrared water index (SWWI)	(R_850_ –R_1650_)/(R_850_ + R_1650_)
Normalized difference infrared index (NDII)	(R_860_ –R_1650_)/(R_860_ + R_1650_)
Normalized difference water index-2130 (NDWI-2130)	(R_858_ –R_2130_)/(R_858_ + R_2130_)
Normalized difference moisture index-1 (NDMI-1; 860;1640;2130)	860 –(R_1640_ –R_2130_) / 860 + (R_1640_ –R_2130_)
Moisture stress index (MSI)	(R_1600_/R_820_)
Ratio spectral index (RWC; RSI; 2264;1321)	(R_2264_/R_1321_)
Normalized difference moisture index-2 (NDMI-2; 2200; 1100)	(R_2200_ –R_1100_)/(R_2200_ + R_1100_)
Normalized difference spectral index (RWC; NDSI)	(R_1222_ –R_2264_)/(R_1222_ + R_2264_)
Normalized difference tillage index (NDTI)	(R_1650_ –R_2215_)/(R_1650_ + R_2215_)
Cellulose absorption index (CAI)	0.5 × (R2031 –R_2211_)–R_2101_

### Statistical analysis

Data on growth and photosynthetic parameters were tested using ANOVAs appropriate for a randomized complete block split-plot design, with irrigation rate as the main factor and cultivar as the split factor. The differences between the mean values of irrigation rates, cultivars, and their interactions were compared using Duncan’s test at the 95% probability level. Pearson’s correlation coefficient matrix was used to determine the relationship among all growth and photosynthetic parameters measured for each irrigation rate. The relationships between different SRIs and TDW and photosynthetic parameters were fitted with linear and non-linear curve-fitting, and the equation with the highest R^2^ was selected as the best equation. The relationships were fitted using Sigma Plot for Windows (version 11.0; Sysat Software Inc., Point Richmond, Chicago, IL, USA).

## Results

### Plant growth parameters

In general, all plant growth parameters [green leaf number per plant (GLN), green leaf area per plant (GLA), and total shoot dry weight per plant (TDW)] were significantly affected by water irrigation rate, cultivar, and their interaction. Averaged over the two seasons, decreases in these growth parameters for moderate water stress (0.75 ETc) and severe water stress (0.50 ETc) relative to the well-watered treatment (1.00 ETc) were 22.2% and 35.5% for GLN, 30.8% and 36.0% for GLA, and 23.3% and 36.5% for TDW, respectively. The differences in these growth parameters between Pavon 76 and Sakha 93 cultivars were not significant, with the exceptions of GLA and TDW in the first season, with the drought-sensitive cultivar Yecora Rojo invariably showing the lowest values, even under 1.00 ETc treatment, when compared with the two other cultivars (Table 3). The values obtained for Pavon 76 under 0.75 and 0.50 ETc or for Sakha 93 under 0.75 ETc were competitive with those for Yecora Rojo under 1.00 ETc. The lowest values for these growth parameters were obtained for Yecora Rojo under either 0.75 or 0.50 ETc treatments, and sometimes for Sakha 93 under 0.50 ETc (Table 3).

**Table 3:** Effects of irrigation rate, cultivar, and their interaction on selected vegetative growth parameters measured at the anthesis growth stage in two growing seasons.

Irrigation rate	2015–2016				2016–2017			
	Cultivars							
	Pavon 76	Sakha 93	Yecora Rojo	Mean	Pavon 76	Sakha 93	Yecora Rojo	Mean
**Number of green leaves per plant**								
**1.00 ETc**	12.79 a	12.70 a	9.90 b	**11.8 A**	13.88 a	13.77 a	10.12 b	**12.6 A**
**0.75 ETc**	9.46 b	9.11 b	6.67 d	**8.4 B**	9.88 bc	8.66 c	6.80 d	**8.4 B**
**0.50 ETc**	9.13 b	8.00 c	5.96 d	**7.7 B**	9.68 bc	8.23 c	5.79 d	**7.9 B**
**Mean**	**10.46A**	**9.94 A**	**7.51 B**		**11.14 A**	**10.22 A**	**7.57 B**	
**Green leaf area per plant (cm**^**-2**^**)**								
**1.00 ETc**	323.7 a	303.6 a	254.2 b	**293.8A**	374.4 a	387.5 a	242.0 bc	**334.6A**
**0.75 ETc**	260.4ab	210.2 c	193.8cd	**221.5B**	280.5 b	235.0bcd	202.1de	**239.2 B**
**0.50 ETc**	223.9bc	175.5 d	139.8 e	**179.7B**	218.8cde	185.2 ef	153.5 f	**185.8C**
**Mean**	**269.4A**	**229.8B**	**195.9 C**		**291.2 A**	**269.2 A**	**199.2 B**	
**Total shoot dry weight per plant (g)**								
**1.00 ETc**	9.05ab	9.20 a	7.14 cd	**8.46 A**	9.43 a	9.39 a	7.41 b	**8.74 A**
**0.75 ETc**	8.03bc	6.58 d	5.15 e	**6.58 B**	7.90 ab	6.71 b	5.20 c	**6.61 B**
**0.50 ETc**	7.02cd	5.07 e	4.28 f	**5.46 B**	6.75 b	5.12 c	4.55 c	**5.47 C**
**Mean**	**8.03 A**	**6.95 B**	**5.52 C**		**8.03 A**	**7.08 A**	**5.72 B**	

Means in columns within cultivar as well as means in rows within irrigation rate followed by the same letter are not significantly different at the 0.05 level according to the Duncan’s test.

The main effect of irrigation rates was tested using the first order interaction, replicate × irrigation rate, as the error term. The main effect of cultivars and the interaction between cultivar and irrigation rate were tested using the second order interaction, replicate × irrigation rate × cultivar, as the error term.

### Photosynthetic parameters

The three photosynthetic parameters [net photosynthesis rate (*P*_*n*_), stomatal conductance (*Gs*), and transpiration rate (*E*)] were also significantly affected by water irrigation rate, cultivar, and their interaction. The three parameters were gradually decreased by decreasing water irrigation rate, with no significant difference between Pavon 76 and Sakha 93 cultivars in *P*_*n*_ and *Gs*, and between Sakha 93 and Yecora Rojo in *E*, regardless of water irrigation rate (Table 4). Under all water irrigation rates, Pavon 76 showed higher values of *P*_*n*_ and *Gs*, and lower values of *E* than Yecora Rojo. Moreover, the values of *P*_*n*_ and *Gs* for Sakha 93 were competitive with those of Pavon 76 under 1.00 and 0.75 ETc, and the values of *E* were occasionally comparable with those of Yecora Rojo under all water irrigation rates (Table 4).

**Table 4:** Effects of irrigation rate, cultivar, and their interaction on photosynthetic parameters measured at the anthesis growth stage in two growing seasons.

Irrigation rate	2015–2016				2016–2017			
	Cultivars							
	Pavon 76	Sakha 93	Yecora Rojo	Mean	Pavon 76	Sakha 93	Yecora Rojo	Mean
**Photosynthetic rate (μmol CO**_**2**_ **m**^**-2**^ **s**^**-1**^**)**								
**1.00 ETc**	19.87 a	20.90 a	16.17 b	**18.98A**	22.73 a	22.57 a	18.20 b	**21.17 A**
**0.75 ETc**	15.47 b	14.37 b	8.53 d	**12.79B**	13.73 c	13.57 c	8.83 d	**12.04 B**
**0.50 ETc**	11.47 c	7.70 d	5.57 e	**8.24 C**	10.82cd	8.20 d	4.67 e	**7.90 C**
**Mean**	**15.60A**	**14.32 A**	**10.09 B**		**15.76 A**	**14.78 A**	**10.57 B**	
**Stomatal conductance (mmol m**^**-2**^ **s**^**-1**^**)**								
**1.00 ETc**	341.9 a	373.1 a	273.4 b	**329.5A**	341.0 b	387.1 a	265.8 cd	**331.3 A**
**0.75 ETc**	286. 7b	277.2 b	218.5 c	**260.8B**	273.3 c	279.8 c	235.8 e	**263.0 B**
**0.50 ETc**	219.4 c	189.3 d	172.6 d	**193.7C**	249.2 e	201.4 f	187.07 f	**212.5 C**
**Mean**	**282.6A**	**279.9 A**	**221.5 B**		**287.8 A**	**289.4 A**	**229.5 B**	
**Transpiration rate (mmol m**^**-2**^ **s**^**-1**^**)**								
**1.00 ETc**	6.53 b	7.93 a	8.50 a	**7.65 A**	6.04 bc	9.03 a	9.37 a	**8.15 A**
**0.75 ETc**	4.38 c	6.33 b	5.80 b	**5.51 B**	4.32 e	6.50 b	5.97 bc	**5.60 B**
**0.50 ETc**	2.75 d	4.79 c	4.43 c	**3.99 C**	3.14 f	5.63 c	5.13 cd	**4.64 C**
**Mean**	**4.55 B**	**6.35 A**	**6.24 A**		**4.50 B**	**7.06 A**	**6.82 A**	

Means in columns within cultivar as well as means in rows within irrigation rate followed by the same letter are not significantly different at the 0.05 level according to the Duncan’s test.

The main effect of irrigation rates was tested using the first order interaction, replicate × irrigation rate, as the error term. The main effect of cultivars and the interaction between cultivar and irrigation rate were tested using the second order interaction, replicate × irrigation rate × cultivar, as the error term.

### Associations between plant growth and photosynthetic parameters under individual irrigation rates

Under each water irrigation rate, the three growth parameters, GLN, GLA, and TDW, showed significant and positive correlations with each other and with *P*_*n*_ and *Gs* (Table 5). Under 1.00 and 0.75 ETc treatments, *E* had a weak negative and non-significant correlation with all growth and photosynthetic parameters. However, under severe water stress (0.50 ETc), *E* was highly and significantly correlated with all parameters. In addition, all parameters showed a stronger relationship with each other under 0.50 ETc compared with 1.00 and 0.75 ETc treatments (Table 5).

**Table 5:** Pearson’s correlation matrix of vegetative growth and photosynthetic parameters across two years and three cultivars (n = 18) under each water irrigation rate.

Parameters	1.00 ETc					
	GLN	GLA	TDW	*P*_*n*_	*Gs*	*E*
**Number of green leaves per plant (GLN)**	1.00	0.92***	0.74**	0.70*	0.88**	-0.51 ns
**Green leaf area per plant (GLA)**		1.00	0.78**	0.80***	0.86**	-0.65 ns
**Total shoot dry weight per plant (TDW)**			1.00	0.66*	0.77**	-0.47 ns
**Photosynthetic rate (*P***_***n***_**)**				1.00	0.76**	-0.51 ns
**Stomatal conductance (*Gs*)**					1.00	-0.30 ns
**Transpiration rate (*E*)**						1.00
	**0.75 ETc**					
**Number of green leaves per plant (GLN)**	1.00	0.77**	0.90***	0.89***	0.92***	-0.52ns
**Green leaf area per plant (GLA)**		1.00	0.78**	0.74*	0.68*	-0.65ns
**Total shoot dry weight per plant (TDW)**			1.00	0.89***	0.80**	-0.65ns
**Photosynthetic rate (*P***_***n***_**)**				1.00	0.90***	-0.34 ns
**Stomatal conductance (*Gs*)**					1.00	-0.33 ns
**Transpiration rate (*E*)**						1.00
	**0.50 ETc**					
**Number of green leaves per plant (GLN)**	1.00	0.94***	0.83**	0.94***	0.81**	-0.69*
**Green leaf area per plant (GLA)**		1.00	0.90***	0.98***	0.86***	-0.77**
**Total shoot dry weight per plant (TDW)**			1.00	0.95***	0.91***	-0.79**
**Photosynthetic rate (*P***_***n***_**)**				1.00	0.91***	-0.74*
**Stomatal conductance (*Gs*)**					1.00	-0.74*
**Transpiration rate (*E*)**						1.00

* Significant at the .05 probability level.

** Significant at the .01 probability level.

*** Significant at the .001 probability level.

ns: not significant.

### Leaf spectral signatures of cultivars under different water irrigation rates

**Fig 1:**
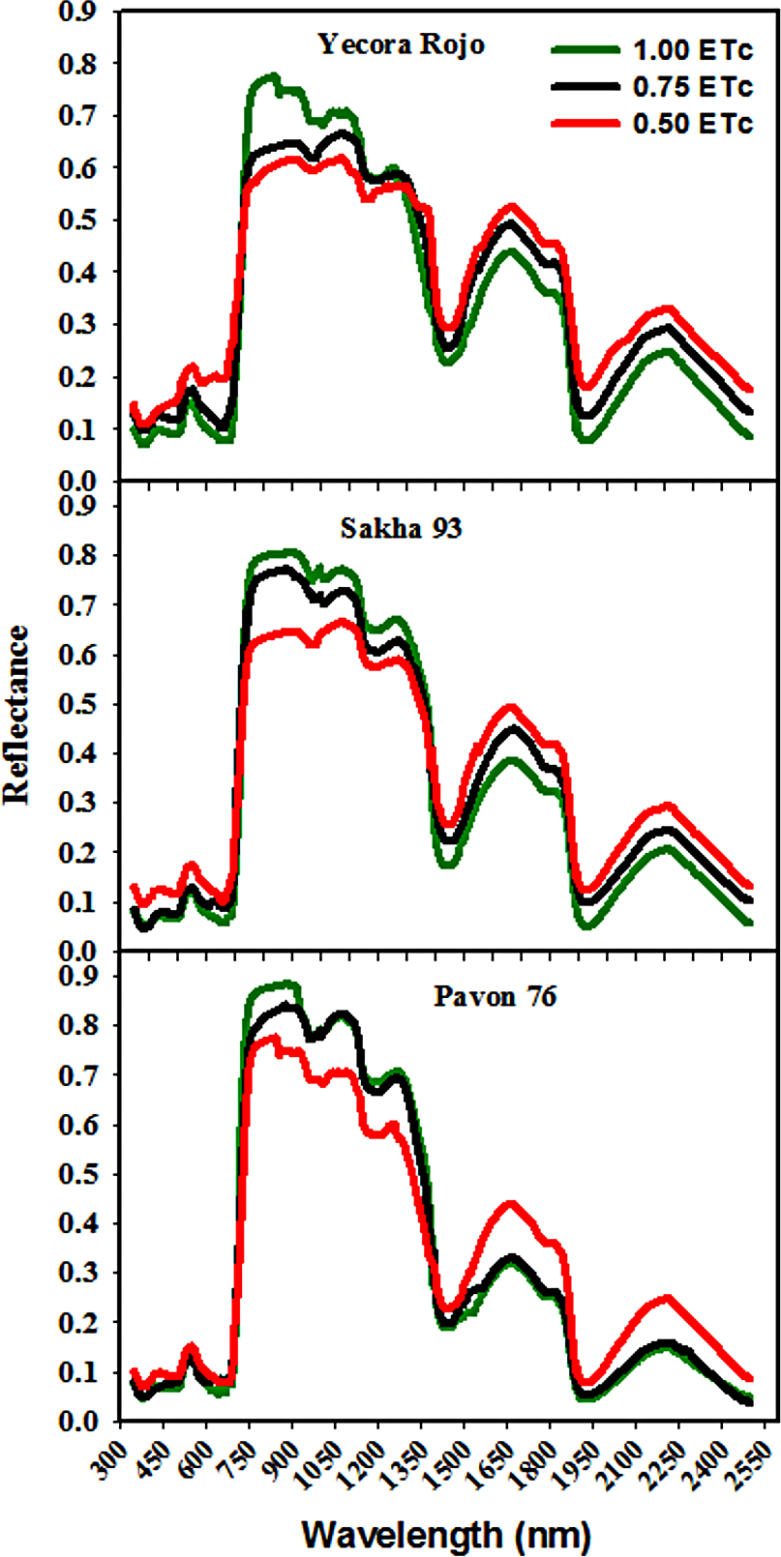
The changes in the shape of reflectance spectra of three cultivars under different irrigation rates in the range between 350 and 2500 nm of spectrum.

Fig 1 shows the behavior of the leaf spectral reflectance of the three cultivars in response to different water irrigation rates throughout the whole electromagnetic spectrum (350 to 2500 nm). Regarding the canopy spectral reflectance in the visible-infrared region (VIS, 400–700 nm), which is related to the leaf chlorophyll content, it can be seen that for most parts of the VIS region of the spectrum, the reflectance curves of the three water irrigation rates are well separated from each other in Yecora Rojo, and that the reflectance values for this cultivar are higher than those of the leaf reflectance values obtained for Pavon 76 and Sakha 93. In the near-infrared (NIR, 700–1300 nm) and shortwave-infrared (SWIR, 1300–2500 nm) regions, in which the magnitude of reflectance is related to the structural discontinuities encountered in the leaf and the absorption characteristics of water and other compounds, respectively, the curves of spectral reflectance for the three water irrigation rates were clearly separated in Yecora Rojo and Sakha 93, whereas the spectral reflectance curves of the 1.00 and 0.75 ETc treatments were very close together in Pavon 76. Further, in all three cultivars, the canopy reflectance for 1.00 ETc in the NIR region was higher than the canopy reflectance for the other two stress treatments (0.75 and 0.50 ETc); the opposite held true in the SWIR region (Fig 1).

### Relationships between different spectral reflectance indices (SRIs) and growth and photosynthetic-related parameters

Thirty-one published SRIs, among which 15, 5, and 11 were selected within the VIS/VIS and NIR/VIS, NIR/NIR, and SWIR/NIR and SWIR/SWIR wavelengths of the spectrum, and are sensitive to changes in leaf pigmentation, structure, and water status, respectively, were regressed with the parameters TDW, *P*_*n*_, *Gs*, and *E*. The equations and determination coefficients (R^2^) of the relationships between these parameters and different SRIs are summarized in Tables 6 and 7.

**Table 6:** The best equations and determination coefficients of the relationships across all data (n = 27) between spectral reflectance indices (SRIs) based on the reflectance in VIS/VIS and NIR/VIS and the parameters (par.) of total shoot dry weight per plant (TDW), net photosynthesis rate (*P*_*n*_), stomatal conductance (*Gs*), and transpiration rate (*E*).

SRIs		Par.	Equations	R^2^	SRIs	Par.	Equations	R^2^
**PRI**	**TDW**		y = 40.6x^0.57^	0.81	**PSND-a**	**TDW**	y = 2.02e^1.64x^	0.67
	***P***_***n***_		y = 308.5x^1.02^	0.80		***P***_***n***_	y = 1.47e^2.91x^	0.65
	***Gs***		y = 1342.6x^0.52^	0.82		***Gs***	y = 91.42e^1.43x^	0.60
	***E***		y = 27.9x + 4.6	0.07		***E***	y = 1.67x + 4.71	0.02
**NPQ**	**TDW**		y = 869.9x^2^ + 181.9 + 13.5	0.72	**PSND-b**	**TDW**	y = 2.14e^1.54x^	0.63
	***P***_***n***_		y = 3423.5x^2^ + 672.3x + 37.4	0.84		***P***_***n***_	y = 1.77e^2.63x^	0.57
	***Gs***		y = 44255x^2^ + 7972.1x + 538.0	0.75		***Gs***	y = 98.34e^1.31x^	0.54
	***E***		y = 2296.0x^2^ + 321.9x + 15.5	0.55		***E***	y = 1.75x + 4.6	0.02
**MCARI**	**TDW**		y = 4.00e^0.40x^	0.88	**PSND-c**	**TDW**	y = 1.89e^1.66x^	0.50
	***P***_***n***_		y = 11.52x^0.73^	0.90		***P***_***n***_	y = 1.35e^2.91x^	0.48
	***Gs***		y = 163.9e^0.34x^	0.84		***Gs***	y = 85.13e^1.46x^	0.47
	***E***		y = 0.96x + 4.7	0.10		***E***	y = 1.58x + 4.72	0.01
**GCI**	**TDW**		y = 3.36e^0.17x^	0.81	**OSAVI**	**TDW**	y = 1.83e^1.76x^	0.73
	***P***_***n***_		y = 3.01x^1.07^	0.81		***P***_***n***_	y = 1.33e^3.03x^	0.67
	***Gs***		y = 138.26e^0.16x^	0.80		***Gs***	y = 86.27e^1.49x^	0.63
	***E***		y = 0.29x + 4.8	0.05		***E***	y = 2.19x + 4.31	0.02
**CTR-2**	**TDW**		y = 2.89x^-0.51^	0.78	**EVI-2**	**TDW**	y = 2.16e^1.40x^	0.78
	***P***_***n***_		y = 28.08e^-3.85x^	0.76		***P***_***n***_	y = 1.69e^2.46x^	0.75
	***Gs***		y = 124.05x^-0.45^	0.72		***Gs***	y = 97.75e^1.20x^	0.69
	***E***		y = -2.89x + 6.6	0.03		***E***	y = 1.77x + 4.50	0.02
**PSSR-a**	**TDW**		y = 3.7765e^0.075x^	0.80	**MTVI**	**TDW**	y = 1.99e^1.23x^	0.84
	***P***_***n***_		y = 2.36x^0.85^	0.76		***P***_***n***_	y = 1.46e^2.18x^	0.81
	***Gs***		y = 158.83e^0.064x^	0.70		***Gs***	y = 92.63e^1.05x^	0.73
	***E***		y = 0.048x + 5.6	0.01		***E***	y = 1.60x + 4.35	0.03
**PSSR-b**	**TDW**		y = 2.55x^0.49^	0.85	**SIPI**	**TDW**	y = 82.89e^-2.43x^	0.40
	***P***_***n***_		y = 2.32x^0.84^	0.79		***P***_***n***_	y = 1728.7e^-4.78x^	0.48
	***Gs***		y = 113.48x^0.42^	0.74		***Gs***	y = 1926.9e^-1.94x^	0.31
	***E***		y = 0.082x + 5.3	0.03		***E***	y = -4.06x + 10.13	0.02
**PSSR-c**	**TDW**		y = 2.20x^0.54^	0.78				
	***P***_***n***_		y = 1.88x^0.92^	0.70				
	***Gs***		y = 97.57x^0.48^	0.72				
	***E***		y = 0.095x + 5.13	0.03				

**Table 7:** The best equations and determination coefficients of the relationships across all data (n = 27) between spectral reflectance indices (SRIs) based on the reflectance in NIR/NIR, SWIR/NIR and SWIR/SWIR and the parameters (par.) of total shoot dry weight per plant (TDW), net photosynthesis rate (*P*_*n*_), stomatal conductance (*Gs*), and transpiration rate (*E*).

SRIs		Par.	Equations	R^2^	SRIs	Par.	Equations	R^2^
**NDVI**	**TDW**		y = 43.4x^2^–25.6x + 8.1	0.90	**NDWI-2130**	**TDW**	y = 2.485e^1.82x^	0.82
	***P***_***n***_		y = 122.9x^2^–67.5x + 14.4	0.86		***P***_***n***_	y = 35.37x^1.65^	0.81
	***Gs***		y = 1638x^2^–101.7x + 333.8	0.86		***Gs***	y = 432.76x^0.807^	0.75
	***E***		y = 11.2x^2^–5.1x + 5.5	0.10		***E***	y = -29.7x^2^ + 34.8x - 3.8	0.12
**WI**	**TDW**		y = 0.085e^4.065x^	0.31	**NDMI-1**	**TDW**	y = 23.105x^2.24^	0.80
	***P***_***n***_		y = 0.0067e^7.01x^	0.29		***P***_***n***_	y = 105.41x^3.87^	0.75
	***Gs***		y = 6.172e^3.48x^	0.28		***Gs***	y = 733.77x^1.88^	0.69
	***E***		y = 2.99x + 2.71	0.03		***E***	y = -85.5x^2^ + 108.3x - 27.8	0.12
**NWI-3**	**TDW**		y = 3.806e^-15.56x^	0.64	**MSI**	**TDW**	y = 15.58e^-1.57x^	0.84
	***P***_***n***_		y = 4.44e^-28.19x^	0.65		***P***_***n***_	y = 55.17e^-2.78x^	0.82
	***Gs***		y = 157.62e^-13.67x^	0.59		***Gs***	y = 533.5e^-1.34x^	0.74
	***E***		y = -18.78x + 5.24	0.02		***E***	y = -2.76x + 7.42	0.05
**NDWI-1240**	**TDW**		y = 27.20x^0.54^	0.63	**RWC;RSI**	**TDW**	y = 14.18e^-2.01x^	0.74
	***P***_***n***_		y = 130.7x^0.91^	0.56		***P***_***n***_	y = 48.44e^-3.66x^	0.76
	***Gs***		y = 856.76x^0.46^	0.56		***Gs***	y = 504.39e^-1.79x^	0.70
	***E***		y = -1798.7x^2^ + 275.6x - 3.3	0.17		***E***	y = -49.8x^2^ + 32.1x + 1.5	0.21
**REM**	**TDW**		y = 3.77e^0.14x^	0.86	**NDMI-2**	**TDW**	y = -16.3x^2^–28.1x - 2.9	0.71
	***P***_***n***_		y = 4.37e^0.25x^	0.88		***P***_***n***_	y = -61.4x^2^–99.0x - 19.9	0.70
	***Gs***		y = 160.07e^0.12x^	0.73		***Gs***	y = -668.6x^2^–1075.8x - 97.4	0.66
	***E***		y = 0.19x^2^–0.99x + 6.2	0.22		***E***	y = -28.1x^2^–31.8x - 2.5	0.14
**RMSI**	**TDW**		y = -1.42x^2^ + 8.5x - 3.5	0.77	**RWC;NDSI**	**TDW**	y = 13.137x^0.91^	0.79
	***P***_***n***_		y = -5.57x^2^ + 30.7x - 22.7	0.72		***P***_***n***_	y = 40.99x^1.62^	0.78
	***Gs***		y = -67.8x^2^ + 359.9x - 150.8	0.67		***Gs***	y = 463.06x^0.79^	0.71
	***E***		y = -2.35x^2^ + 9.9x - 3.8	0.16		***E***	y = -41.6x^2^ + 45.3x - 5.7	0.17
**SWWI**	**TDW**		y = 12.09x^0.42^	0.84	**NDTI**	**TDW**	y = 29.007x^1.17^	0.54
	***P***_***n***_		y = 35.71x^0.77^	0.84		***P***_***n***_	y = 133.97x^1.90^	0.45
	***Gs***		y = 426.49x^0.36^	0.72		***Gs***	y = 906.52x^0.99^	0.48
	***E***		y = -32.4x^2^ + 21.1x + 3.1	0.13		***E***	y = -121.5x^2^ + 70.9x - 4.1	0.02
**NDII**	**TDW**		y = 12.07x^0.42^	0.81	**CAI**	**TDW**	y = 16.91e^3.84x^	0.73
	***P***_***n***_		y = 36.22x^0.77^	0.84		***P***_***n***_	y = 61.072e^6.62x^	0.68
	***Gs***		y = 433.21x^0.37^	0.75		***Gs***	y = 565.81e^3.24x^	0.63
	***E***		y = -34.23x^2^ + 22.45x + 2.8	0.14		***E***	y = -141x^2^–61.1x - 0.15	0.11

These relationships were obtained after the data for the replications, water irrigation rates, and cultivars were pooled together. On the basis of the R^2^ values, exponential and power equations were the best models describing the relationships between SRIs and TDW, *P*_*n*_, and *Gs*, with the exception of the normalized phaeophytinization index (NPQ) in the VIS/VIS region, the normalized difference vegetation index (NDVI) in the NIR/NIR region, and the reciprocal of the moisture stress index (RMSI) and the normalized difference moisture index-1 (NDMI-1) in the SWIR/NIR region, which all showed second-order relationships. The values of R^2^ for these relationships ranged from 0.40 to 0.88, from 0.31 to 0.90, and from 0.54 to 0.84 for TDW; from 0.48 to 0.90, from 0.29 to 0.88, and from 0.45 to 0.84 for *P*_*n*_; and from 0.31 to 0.84, from 0.28 to 0.86, and from 0.48 to 0.75 for *Gs*, with the SRIs based on the reflectance in VIS/VIS and NIR/VIS, NIR/NIR, and SWIR/NIR wavelengths of the spectrum, respectively. However, with the exception of NPQ, which showed a moderate relationship (R^2^ ≈ 0.55, P < 0.05), none of the SRIs showed a relationship with *E* (Tables 6 and 7).

**Fig 2:**
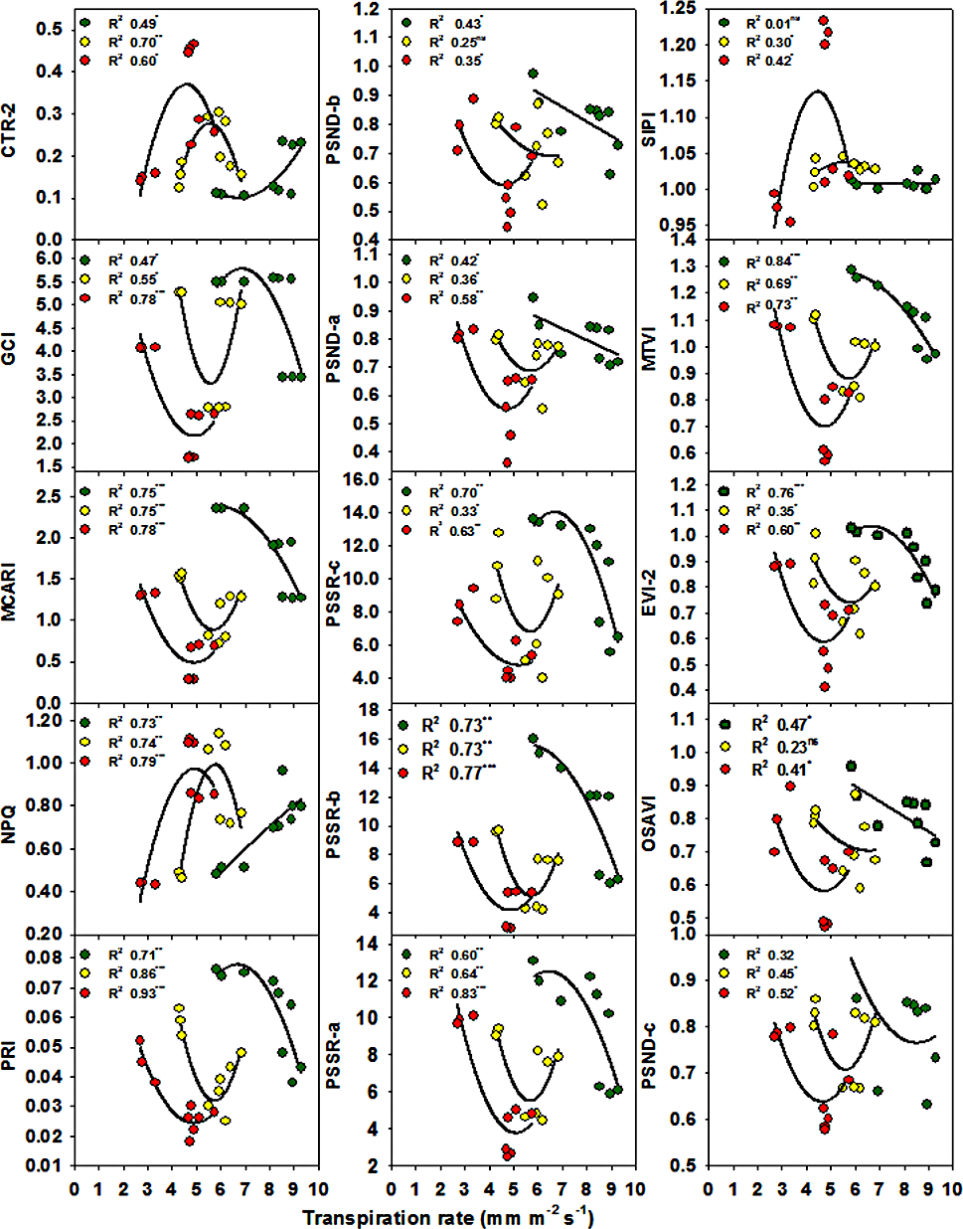
Relationships between transpiration rates and spectral reflectance indices based on the reflectance in VIS/VIS and NIR/VIS regions for 1.00 ETc (green), 0.75 ETc (yellow) and 0.50 ETc (red) treatments. Data correspond to the three cultivars and three replications for each irrigation rate. *,**, ***Significant at the .05, .01, and .001 probability levels, respectively, and ns: not significant.

**Fig 3:**
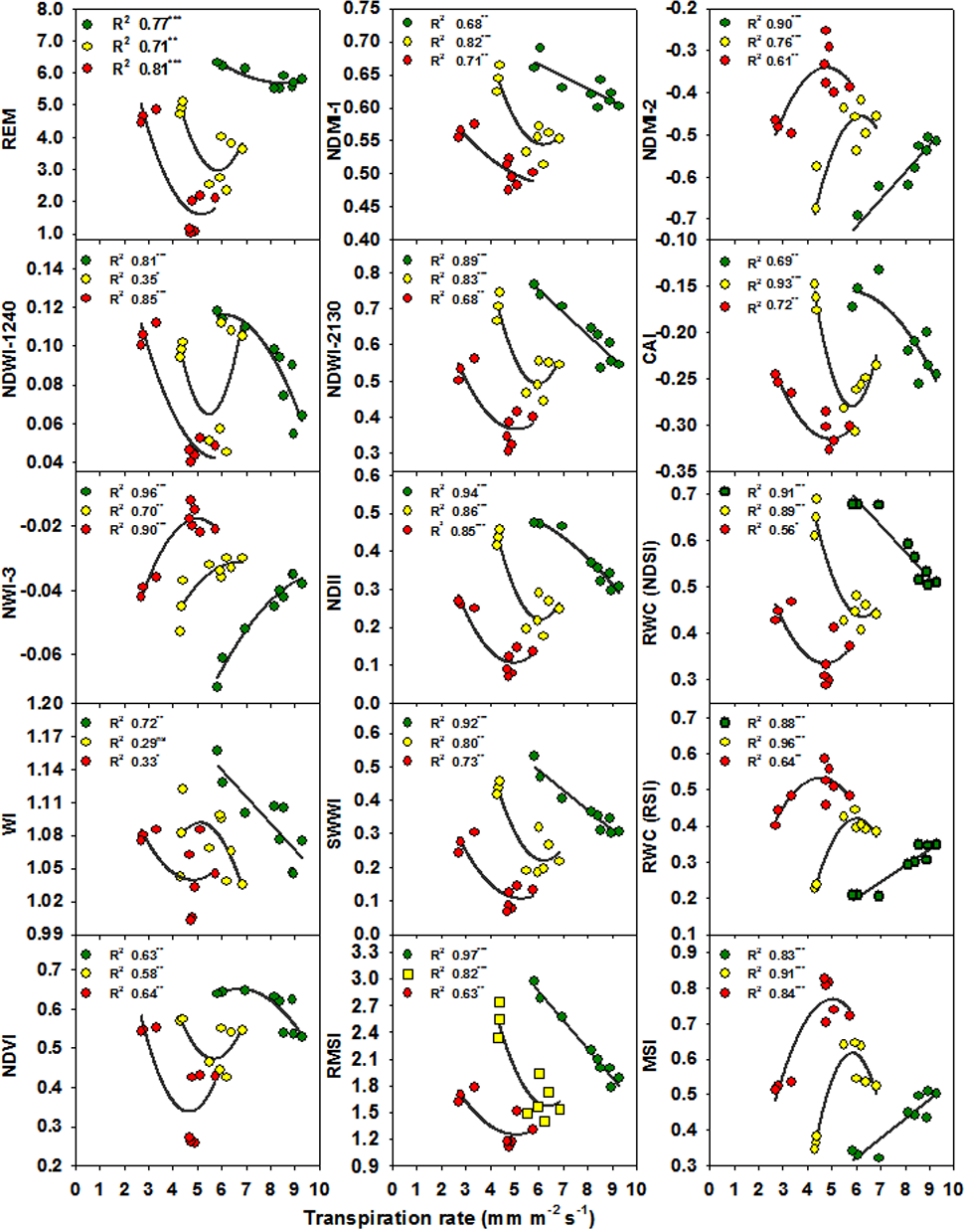
Relationships between transpiration rates and spectral reflectance indices based on the reflectance in NIR/NIR, SWIR/NIR and SWIR/SWIR regions for 1.00 ETc (green), 0.75 ETc (yellow) and 0.50 ETc (red) treatments. Data correspond to the three cultivars and three replications for each irrigation rate. *,**, ***Significant at the .05, .01, and .001 probability levels, respectively, and ns: not significant.

The relationships between the different SRIs and growth and photosynthetic parameters for each water irrigation rate are presented in Figs 2 and 3 and S1–S6 Figs. Considering the R^2^ values, most of the SRIs had curvilinear relationships (a few had linear relationships) with the four parameters under each water irrigation rate. Interestingly, although all SRIs, except NPQ, were not significantly related to *E* when all the data of irrigation rates were pooled together, 21, 18, and 16 of the 31 SRIs showed a coefficient of determination with *E* higher than 0.65 under 1.00, 0.75, and 0.50 ETc treatments, respectively (Figs 2 and 3). Regarding the relationships between the SRIs and TDW, *P*_*n*_, and *Gs*, it is noteworthy that these relationships fitted better under 0.75 and 0.50 ET than under 1.00 ET, and this was more evident for TDW and *P*_*n*_, whereas for Gs, the values of R^2^ for the relationships with SRIs were almost the same under the three water irrigation rates (S1–S6 Figs).

## Discussion

### Interpreting the response of morphophysiological parameters to water irrigation rates and the relationship between each other

Maintaining plant growth and development under limited water availability could be considered as an important mechanism in the adaptation of plants to water deficit stress and could also be used as an accurate indicator for the management of precision irrigation in irrigated arid regions because, in most cases, specific plant growth parameters, such as green leaf number (GLN), green leaf area (GLA) and total shoot dry weight (TDW), and photosynthetic efficiency are directly proportional to each other. As observed in the present study, the three photosynthetic parameters [net photosynthesis rate (*P*_*n*_), stomatal conductance (*Gs*), and transpiration rate (*E*)] gradually decreased with decreasing water irrigation rates, and were accompanied by parallel reductions in plant growth parameters (Tables 3 and 4). Thus, a significant correlation was observed between these photosynthetic parameters and GLN, GLA, and TDW, particularly under the 0.50 ETc treatment (Table 5), which indicates the close relationship between plant growth parameters and photosynthetic efficiency under water deficit stress. In contrast, some studies have demonstrated that there is a weak relationship between *P*_*n*_ and plant growth under water deficit stress, where the water stress results in a strong reduction in plant growth, but does not significantly affect the rate of photosynthesis [48–52]. However, our findings are in agreement with those of other studies in which a strong association was observed between plant growth and photosynthetic properties [6, 53, 54]. The differences between these studies could be related to genotypic differences in mechanisms of adaption to various water deficit stresses, where some genotypes can adapt to this stress by reducing their growth, while simultaneously maintaining photosynthetic rates [49, 52]. However, other genotypes could have higher stomatal conductance under water deficit stress, which results in high rates of both photosynthesis and transpiration, and facilitates greater CO_2_ fixation per unit leaf area [55]. However, a significant increase in transpiration rate and non-conservative use of available soil water during a water stress period will result in decreases in plant growth, particularly for genotypes that do not have an efficient mechanism for capturing water from the deeper soil profiles. Thus, this eventually leads to a significant decrease in photosynthetic efficiency, which coincides with a decrease in plant growth. This pattern was confirmed in the present study, in which transpiration rate showed stronger and negative correlations with the growth parameters, *P*_*n*_ and *Gs* under the 0.50 ETc treatment compared to the 1.00 and 0.75 ETc treatments (Table 5). This may be the reason why growth and photosynthetic parameters were correlated and simultaneously reduced. The negative correlation between Gs and *E* under each irrigation treatment indicate that different stomatal characteristics such as stomatal density, and stomatal size (length and width of stomata) as well as environmental conditions may important factors influencing stomatal conductance and transpiration rate in plants [56].

### Interpreting canopy reflectance spectra of cultivars under different water irrigation rates

The second main objective of this study was to assess whether spectrum optical properties have potential use in monitoring the growth and photosynthetic properties of wheat cultivars under different water irrigation rates. The results shown in Fig 1 indicate that there are subtle differences in the shape of canopy reflectance curves for each examined cultivar under the three water irrigation rates in the three parts of the spectrum [visible (VIS; 400–700 nm), near-infrared (NIR; 700–1300 nm), and shortwave-infrared (SWIR; 1300–2500 nm) regions], which effectively characterize differences in the spectral signatures of leaf photosynthetic pigmentation, vegetation structure, and vegetation water content, respectively [1, 20, 23, 29, 32, 34, 42, 43]. The results present in Fig 1 also reveals that it is possible to capture the effect of different water irrigation rates on the growth and photosynthetic properties of wheat cultivars in terms of their spectral signature in the three parts of the spectrum of wavelengths. These results are consistent with those of [42, 57–59], who pointed out that the changes in canopy reflectance properties induced by water stress can be detected in the full range of wavelengths of the spectrum (400–2500 nm), which indicates that it is possible to assess the growth and photosynthetic properties of wheat cultivars under different irrigation rates over a wide range of the spectrum. This can be explained by the fact that the influence of water deficit stress on canopy reflectance spectra includes both direct and indirect effects. The direct effects relate to the spectral properties of the canopy’s water content itself, which gives rise to changes in reflectance in the SWIR range because of a lack of absorption by water. The indirect effects, which are manifested by changes in reflectance in the VIS and NIR ranges, are associated with leaf and canopy properties, such as leaf pigments, and leaf structure and scattering, respectively, which change under variable water stress treatments [58]. Bayat et al. [42] reported that, in grass, the percentage of indirect effects is less than that of direct effects during the early responses to water stress (11 days after induction of water stress), whereas the opposite trend was observed at a late stage of water stress (36 days after induction of water stress). Therefore, in the present study, we continued to explore the potential of several published spectral reflectance indices (SRIs), which combine the reflectance of wavelengths in three parts of the spectrum and are able to capture a broad variation in dominant vegetation properties, as a proxy tool for effective assessment of the growth and photosynthetic properties of wheat cultivars under variable water irrigation rates, non-destructively and in real-time under field conditions.

### Assessment of growth and photosynthetic efficiency under different water irrigation rates using a wide range of spectral reflectance indices (SRIs)

From the results of the regression analysis, we noted that, in most cases, the best-fit equations for assessment of TDW, *P*_*n*_, and *Gs* by SRIs were power and exponential curves (Tables 6 and 7). The reason for these curvilinear relationships may be that the three cultivars exhibited different responses to different water irrigation rates in their growth and photosynthetic properties, and that the sensitivity of different SRIs to monitor canopy characteristics might be dependent on the degree of change in these characteristics under different water irrigation rates. In this study, the cultivar Sakha 93 showed values for *P*_*n*_ and *Gs* comparable to those of Pavon 76, although the *E* and TDW values of Sakha 93 were significantly higher and lower, respectively, than those of Pavon 76 under 0.75 and 0.50 ETc (Tables 3 and 4). Therefore, the two cultivars differed from each other in spectral properties in the NIR and SWIR regions, whereas in the VIS region, the spectral properties of both cultivars were very similar (Fig 1). These results indicate that the relationships between vegetative parameters and SRIs are highly genotype-dependent. This assumption is consistent with the findings of Zhang et al. [60], who affirmed that if the relationship between SRIs and the chlorophyll contents of different rice cultivars differing in chlorophyll contents were fitted, none of the best-fit equations for assessment of chlorophyll *a*, chlorophyll *b*, and total chlorophyll contents are linear equations; they are polynomial, exponential, and power.

Most of the SRIs examined in the present study, which are based on VIS-vs.-VIS, NIR-vs.-VIS, NIR-vs.-NIR, NIR-vs.-SWIR or SWIR-vs.-SWIR wavelengths, showed a strong relationship with TDW, *P*_*n*_, and *Gs*, whereas they failed to track the changes in *E* (Tables 6 and 7). These results indicate that most of the SRIs associated with photosynthetic pigment concentrations, leaf structure, and plant water status could be used as rapid and non-destructive tools for monitoring the growth and photosynthetic properties of wheat cultivars under a range of water irrigation rates. The results of this study also indicate that the SRIs that utilize the reflectance of VIS-vs.-VIS bands, such as photochemical reflectance index (PRI) and normalized phaeophytinization index (NPQ), or NIR-vs.-VIS bands, such as green chlorophyll index (GCI), modified chlorophyll absorption ratio index (MCARI), modified transformed vegetation index (MTVI), pigment specific simple ratio-*a*, *b* and *c* (PSSR-*a*, PSSR-*b* and PSSR-*c*), and enhanced vegetation index-2 (EVI-2), showed the highest predictive performance for assessments of TDW, *P*_*n*_, and *Gs* (R^2^ ≥ 0.70) like the SRIs selected within the NIR-vs.-NIR, NIR-vs.-SWIR, or SWIR-vs.-SWIR spectral regions (Tables 6 and 7). These findings indicate that the SRIs in the formula of normalized differences spectral indices (NDSI) and based on VIS-vs.-VIS or NIR-vs.-VIS bands are sufficient and suitable for assessing the growth and photosynthetic properties of wheat cultivars under a wide range of water irrigation rates. These findings also suggest that wavelengths in the VIS and NIR ranges that are not exploited in the published NDSI formulae may offer considerable potential for tracking changes in the growth and photosynthetic efficiency of wheat crops under different irrigation rates. The utility of SRIs that incorporate VIS and NIR wavelengths to improve the accuracy in estimating the growth and photosynthetic properties of wheat may be related to the fact that the response of plants to water stress in the VIS range is possibly associated with a stress-induced decline in the concentrations of leaf photosynthetic pigments. This could therefore represent a non-stomatal limiting factor in addition to stomatal limitation of photosynthetic efficiency under water stress. However, the responses of plants measured in the NIR range may be attributable to an indirect effect of water stress via changes in the structural properties and scattering at the leaf and canopy scales, which showed significant differences between non-stressed and stressed canopies and between the examined cultivars. Therefore, most of the SRIs based on VIS-vs.-VIS and/or NIR-vs.-VIS wavelengths, and which showed strong relationships with TDW, *P*_*n*_ and *Gs* in this study, have in previous studies been considered as proxies of photosynthetic pigment concentrations, gas exchange variables, biomass accumulation, and plant water status under stressed and normal conditions [21, 23, 34, 57, 61–63].

In this study, the SRIs based on NIR-vs.-SWIR wavelengths, and which are indicators of the vegetative water status such as moisture stress index (MSI), normalized difference water index-2130 (NDWI-2130), shortwave infrared water index (SWWI), normalized difference moisture index-1 (NDMI-1), and normalized difference moisture index-2 (NDMI-2), showed higher predictive power in the assessment of TDW, *P*_*n*_, and *Gs* (R^2^ ranging from 0.70 to 0.84) than the SRIs that are based only on NIR wavelengths (NIR-vs.-NIR), and which have been most commonly used in previous studies [42, 58, 64] for the assessment of the plant water status, such as the normalized water index-3 (NWI-3) and the normalized difference water index-1240 (NDWI-1240) (R^2^ ranging from 0.57 to 0.65) (Tables 6 and 7). The reason for this finding is that the former SRIs are based on one type of sensitive wavelength (SWIR) together with one type of insensitive wavelength (NIR) as a reference to interpret changes in vegetative water content. The reflectance in the SWIR region is an indicator of liquid water molecules in vegetative tissues that interact with the incoming solar radiation, whereas the reflectance in the NIR region is related to vegetation structure, emissivity, and energy balance [42, 58, 64]. Therefore, the ratio between the sensitive and insensitive wavelengths can minimize variations in the properties of canopy scattering [65]. However, NIR-based SRIs, similar to NWI-3 and NDWI-1240, do not detect vegetative water content directly but do detect the vegetative structures and leaf anatomy that change with hydration and water stress [22]. Taken together, our results revealed that the SRIs formulated using SWIR and NIR wavelengths performed better than those SRIs based only on NIR wavelengths in assessing the growth and photosynthetic efficiency of wheat cultivars under a wide range of water irrigation. This is because such SRIs are more sensitive to changes in the vegetative water content, which is an indicator of the growth and photosynthetic efficiency of plants under water stress, than other SRIs that incorporate only NIR wavelengths. These findings are in full agreement with Bayat et al. [42] and Ranjan [43], who concluded that in wheat and grass plants, respectively, the SRIs that incorporate SWIR and NIR wavelengths performed better at detecting the vegetative water status compared with the SRIs based only on NIR wavelengths. However, our results contrast with those reported by Lobos et al. [22, 29], who found that SRIs formulated using only NIR wavelengths, such as NWI-3, are sufficient to assess genotypic differences in wheat with respect to vegetative water status. They explained this utility of NIR wavelengths for estimating water content by suggesting that NIR wavelengths penetrate deeper into the canopy. However, reflectance in the NIR region may not only be associated with the amount of water stored in the leaf cells but may also reflect structural changes in leaf and canopy characteristics that are induced by water stress [19, 66] Such results suggest that there is still scope for examining further SRIs using SWIR and NIR wavelengths.

Interestingly, the results of the present study also showed that all the SRIs examined failed to track the changes in *E* when all the data of water irrigation rates were pooled together (Tables 6 and 7). However, when the regressions of SRIs with *E* were compared for each water irrigation rate, the relationships between all of the SRIs and *E* had high determination coefficients (Figs 2 and 3); the few exceptions being the SRIs such as structure insensitive pigment index (SIPI), NPQ, pigment specific normalized difference-a (PSND-*a*), pigment specific normalized difference-b (PSND-*b*), and pigment specific normalized difference-c (PSND-*c*) that have been found in previous studies to be the best descriptors of leaf pigment concentrations (Figs 2 and 3). Furthermore, the values of the determination coefficients for the relationships between most of SRIs and TDW, *P*_*n*_, and *Gs* were very high under 0.75 and 0.50 ETc treatments when compared with the results obtained when all the data of water irrigation rates were pooled together (S1–S6 Figs). These results indicate that it is better to assess *E* or other parameters related to growth and photosynthetic properties under individual irrigation rates when different cultivars are evaluated under a wide range of irrigation rates. An explanation for these results may be that the different cultivars might exhibit contrasting mechanisms of drought tolerance under different water irrigation rates and the fact that variations in transpiration rate are generally associated with changes in temperature, soil moisture content, and canopy structure among cultivars [61].

In conclusion, the results of this study clearly demonstrate that in order to conserve irrigation water and increase its use efficiency in irrigated arid regions, there is an urgent need to assess multiple morphophysiological parameters simultaneously. Assessing growth and photosynthetic properties of different wheat cultivars under various water irrigation rates using traditional methods is either an unrepresentative tool or necessitates destructive and time-consuming measurements. The hyperspectral reflectance sensing technique is a rapid, non-destructive, and cost-efficient alternative tool for simultaneously monitoring changes in growth and photosynthetic properties via the detection of changes in the spectral signature of plant canopies. The VIS/VIS- and NIR/VIS-based indices show relationships with growth and photosynthetic parameters similar to those of indices based on NIR/NIR or SWIR/NIR. Almost all the SRIs examined in this study assessed growth and photosynthetic parameters, including *E*, more efficiently when the regressions were analyzed for each water irrigation rate.

## Supporting information

S1 Fig Relationships between total shoot dry weight and spectral reflectance indices based on the reflectance in VIS/VIS and NIR/VIS regions for 1.00 ETc (green), 0.75 ETc (yellow) and 0.50 ETc (red) treatments. Data correspond to the three cultivars and three replications for each irrigation rate. *,**, ***Significant at the .05, .01, and .001 probability levels, respectively, and ns: not significant.
(TIF)

S2 Fig Relationships between total shoot dry weight and spectral reflectance indices based on the reflectance in NIR/NIR, SWIR/NIR and SWIR/SWIR regions for 1.00 ETc (green), 0.75 ETc (yellow) and 0.50 ETc (red) treatments. Data correspond to the three cultivars and three replications for each irrigation rate. *,**, ***Significant at the .05, .01, and .001 probability levels, respectively, and ns: not significant.
(TIF)

S3 Fig Relationships between net photosynthesis rate and spectral reflectance indices based on the reflectance in VIS/VIS and NIR/VIS regions for 1.00 ETc (green), 0.75 ETc (yellow) and 0.50 ETc (red) treatments. Data correspond to the three cultivars and three replications for each irrigation rate. *,**, ***Significant at the .05, .01, and .001 probability levels, respectively, and ns: not significant.
(TIF)

S4 Fig Relationships between net photosynthesis rate and spectral reflectance indices based on the reflectance in NIR/NIR, SWIR/NIR and SWIR/SWIR regions for 1.00 ETc (green), 0.75 ETc (yellow) and 0.50 ETc (red) treatments. Data correspond to the three cultivars and three replications for each irrigation rate. *,**, ***Significant at the .05, .01, and .001 probability levels, respectively, and ns: not significant.
(TIF)

S5 Fig Relationships between stomatal conductance and spectral reflectance indices based on the reflectance in VIS/VIS and NIR/VIS regions for 1.00 ETc (green), 0.75 ETc (yellow) and 0.50 ETc (red) treatments. Data correspond to the three cultivars and three replications for each irrigation rate. *,**, ***Significant at the .05, .01, and .001 probability levels, respectively, and ns: not significant.
(TIF)

S6 Fig Relationships between stomatal conductance and spectral reflectance indices based on the reflectance in NIR/NIR, SWIR/NIR and SWIR/SWIR regions for 1.00 ETc (green), 0.75 ETc (yellow) and 0.50 ETc (red) treatments. Data correspond to the three cultivars and three replications for each irrigation rate. *,**, ***Significant at the .05, .01, and .001 probability levels, respectively, and ns: not significant.
(TIF)
