# Supplementary figures and images for: Hyperspectral reflectance sensing to assess the growth and photosynthetic properties of wheat cultivars exposed to different irrigation rates in an irrigated arid region
Source: PLoS One. 2017 Aug 22;12(8):e0183262. doi: 10.1371/journal.pone.0183262 (PMC5567659; doi:10.1371/journal.pone.0183262)

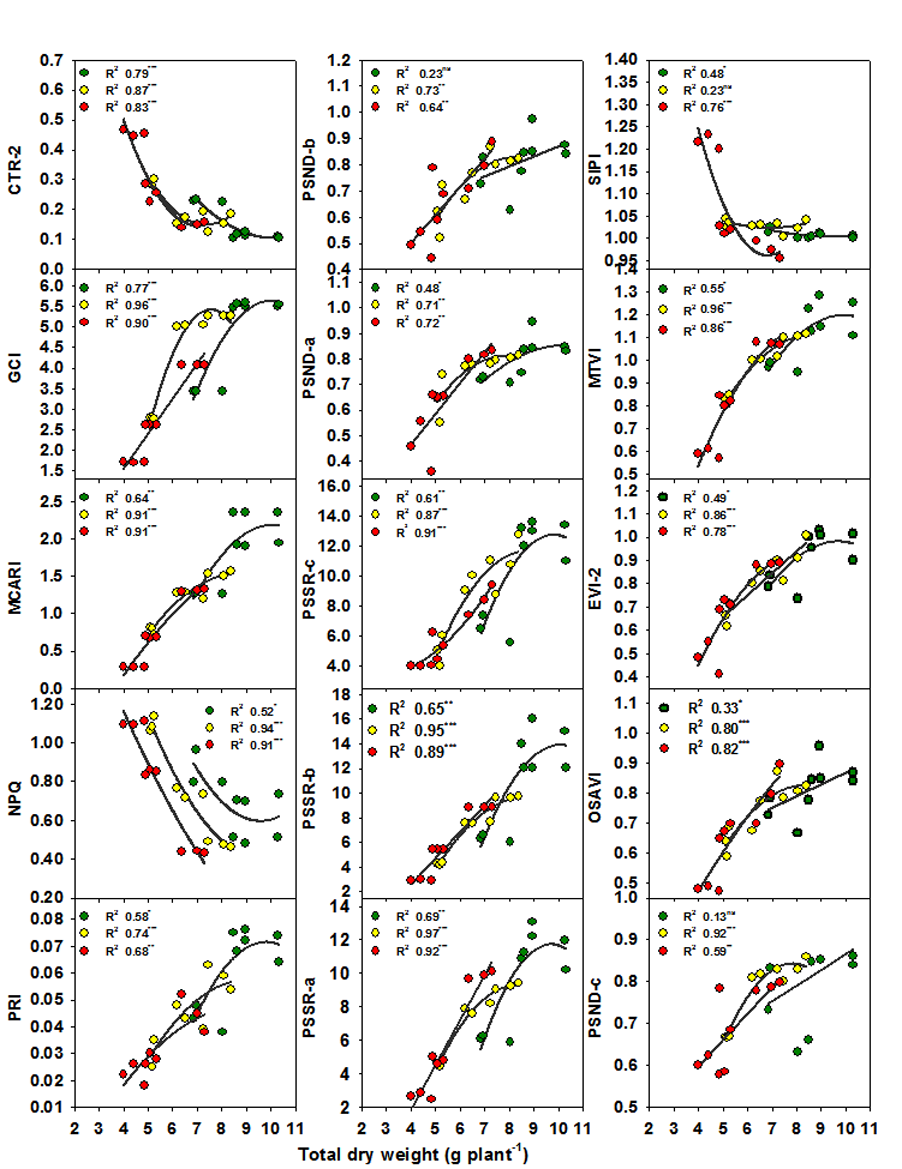

Supplement: S1 Fig — Data correspond to the three cultivars and three replications for each irrigation rate. *,**, ***Significant at the .05, .01, and .001 probability levels, respectively, and ns: not significant. (TIF) [file pone.0183262.s001.tif]

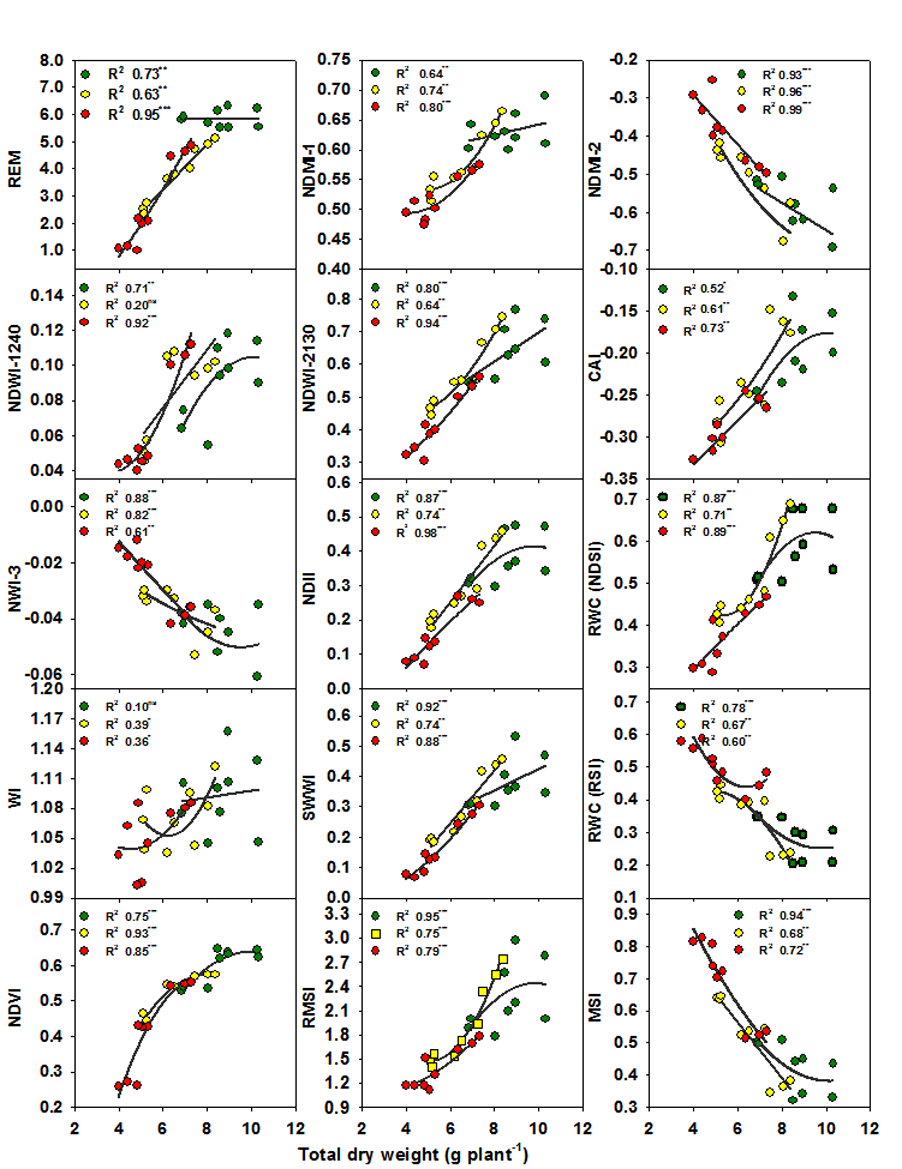

Supplement: S2 Fig — Data correspond to the three cultivars and three replications for each irrigation rate. *,**, ***Significant at the .05, .01, and .001 probability levels, respectively, and ns: not significant. (TIF) [file pone.0183262.s002.tif]

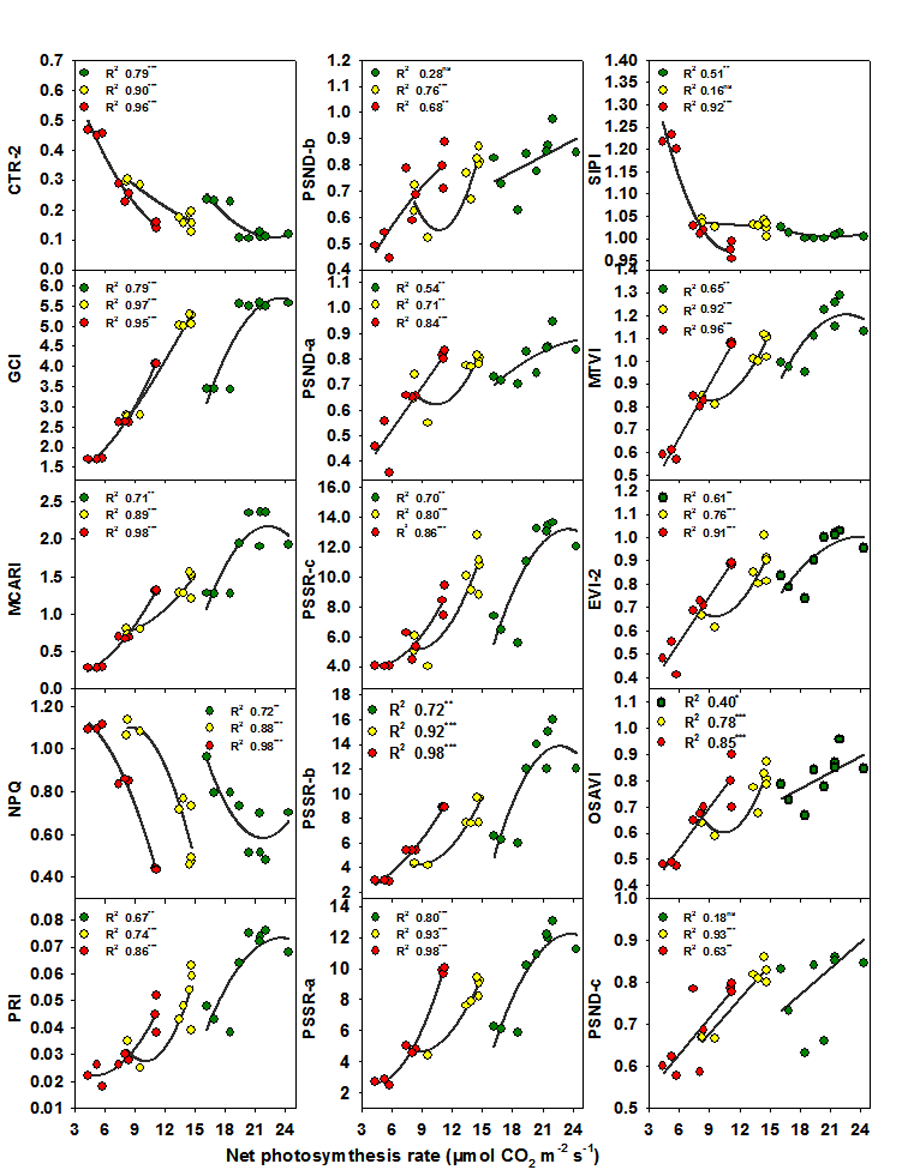

Supplement: S3 Fig — Data correspond to the three cultivars and three replications for each irrigation rate. *,**, ***Significant at the .05, .01, and .001 probability levels, respectively, and ns: not significant. (TIF) [file pone.0183262.s003.tif]

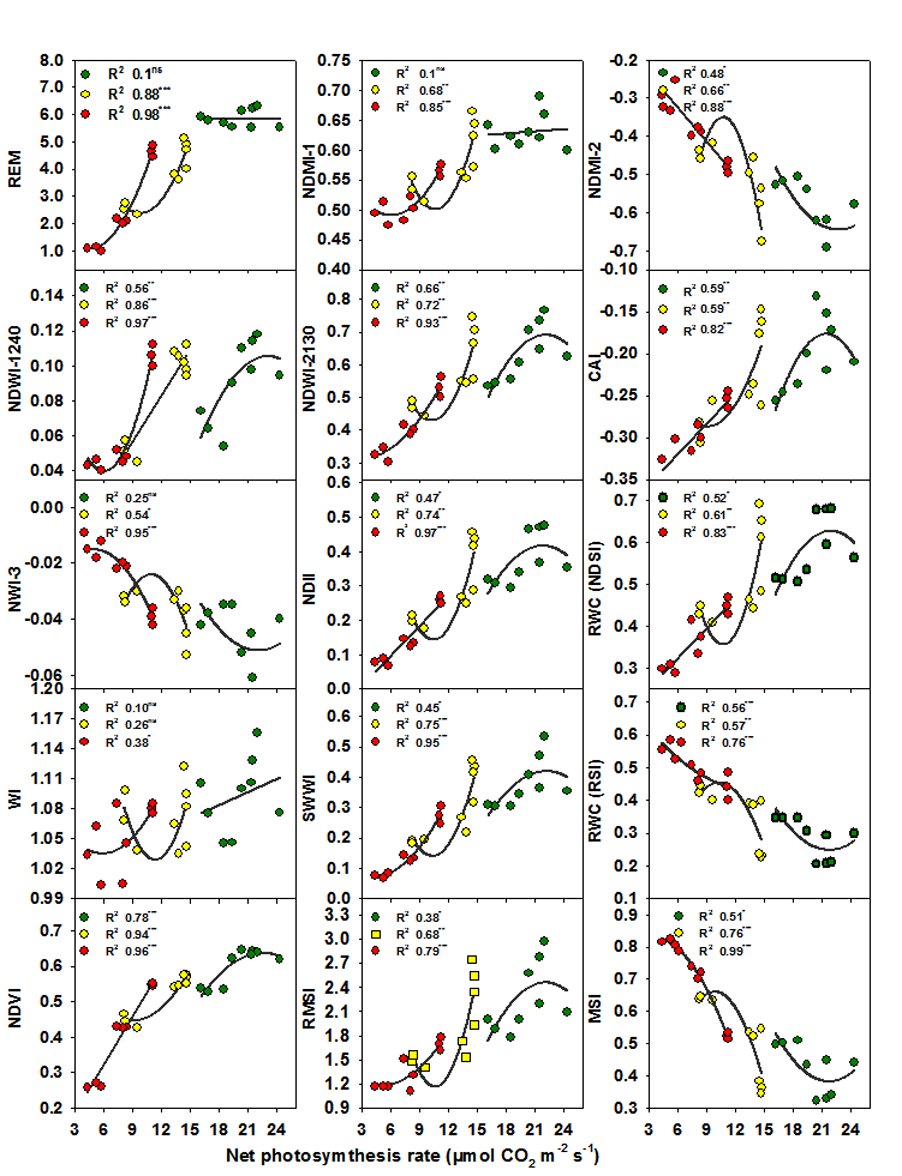

Supplement: S4 Fig — Data correspond to the three cultivars and three replications for each irrigation rate. *,**, ***Significant at the .05, .01, and .001 probability levels, respectively, and ns: not significant. (TIF) [file pone.0183262.s004.tif]

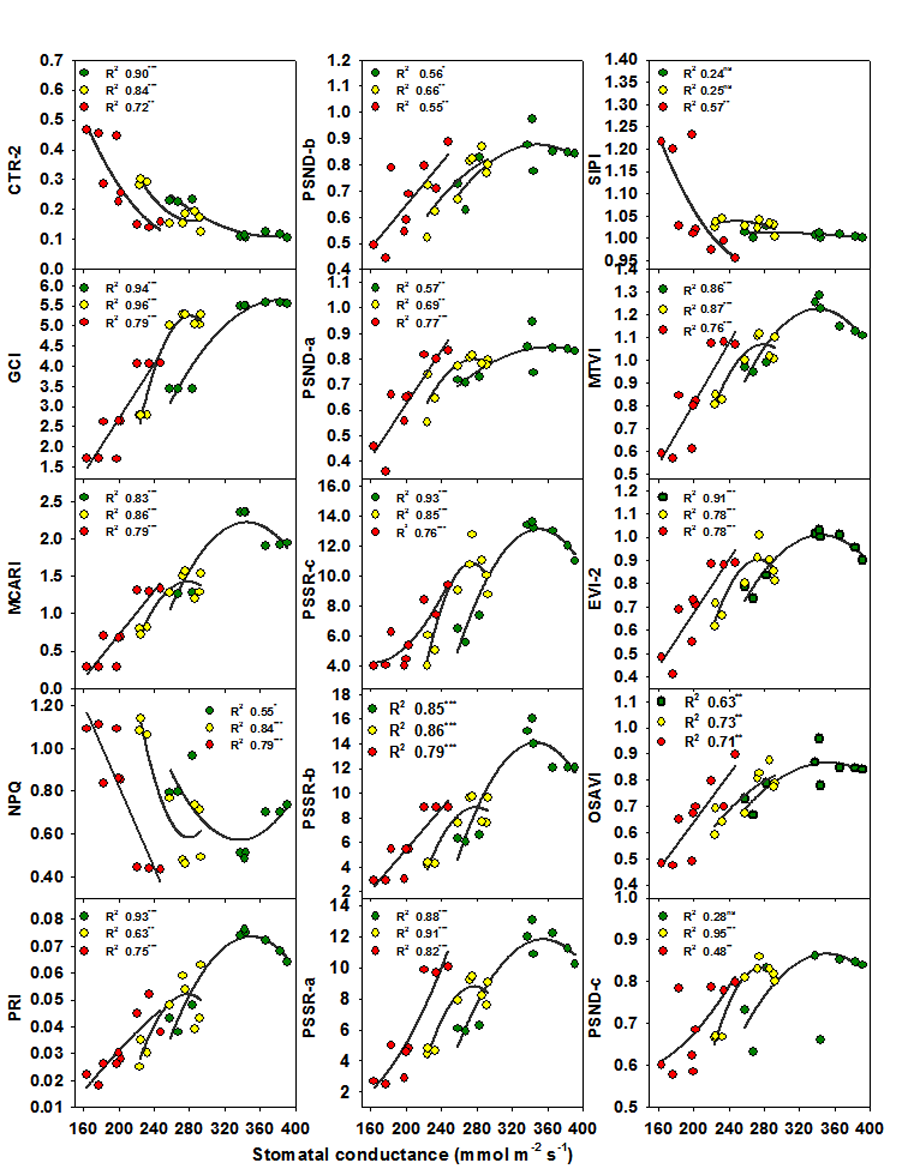

Supplement: S5 Fig — Data correspond to the three cultivars and three replications for each irrigation rate. *,**, ***Significant at the .05, .01, and .001 probability levels, respectively, and ns: not significant. (TIF) [file pone.0183262.s005.tif]

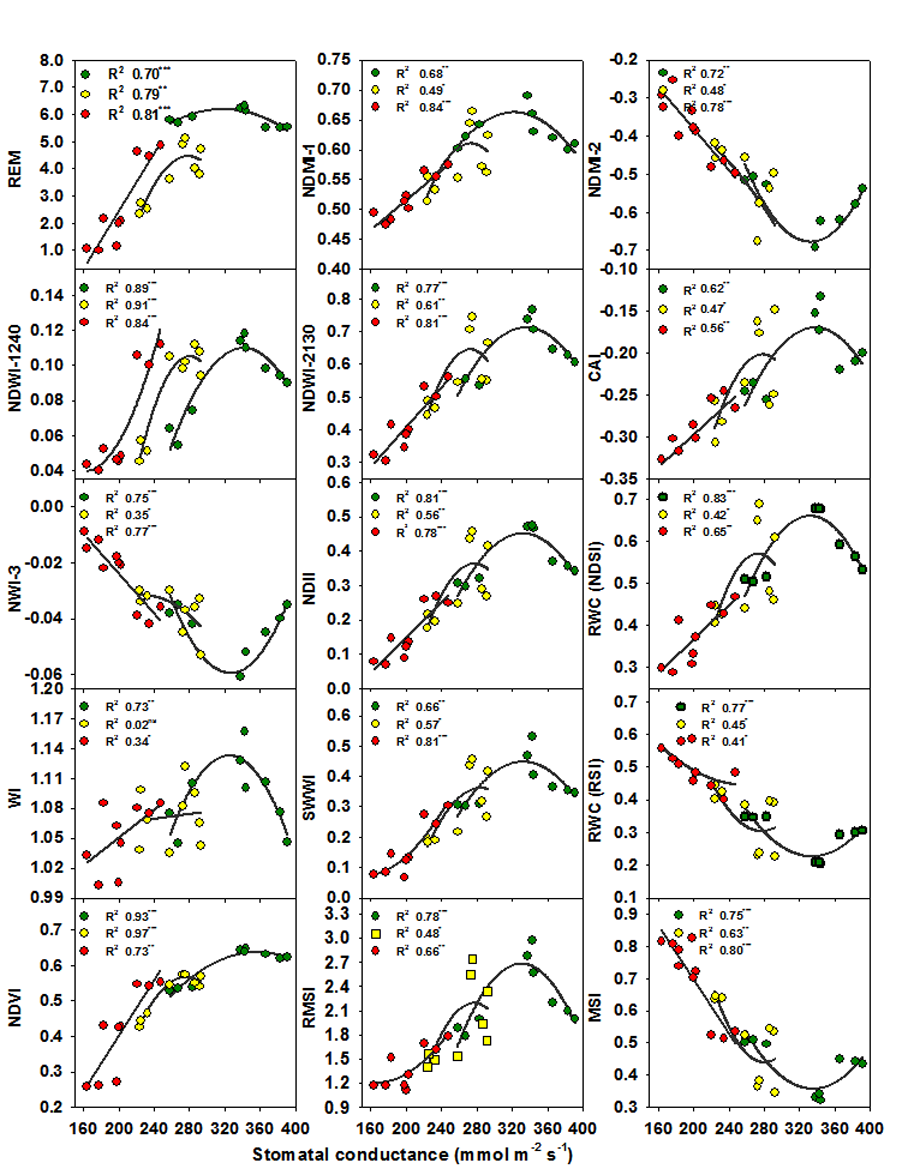

Supplement: S6 Fig — Data correspond to the three cultivars and three replications for each irrigation rate. *,**, ***Significant at the .05, .01, and .001 probability levels, respectively, and ns: not significant. (TIF) [file pone.0183262.s006.tif]
